# Supplementary material for: The population structure of invasive Lantana camara is shaped by its mating system
Source: eLife. 2026 Jul 23;14:RP104988. doi: 10.7554/eLife.104988 (PMC13395457; doi:10.7554/eLife.104988)
Supplement: Supplementary file 1. [file elife-104988-supp1.docx]

**Supplementary material of the paper “*The population structure of invasive Lantana camara is shaped by its mating system”***

P. Praveen, Rajesh Gopal, Uma Ramakrishnan

Table A Sampling locations

| **No** | **location** | **Abbreviations** |
| --- | --- | --- |
| 1 | Andaman | AND |
| 2 | Arunachal | ARU |
| 3 | Bangalore | BAN |
| 4 | Bhuvaneswar | BHU |
| 5 | BTR | BTR |
| 6 | Cherrapunji | CHI |
| 7 | Delhi | DEL |
| 8 | Gudalur | GUD |
| 9 | Hyderabd | HYD |
| 10 | Indore | IND |
| 11 | Jaipur | JAI |
| 12 | Jamnagar | JAM |
| 13 | Jodhpur | JOD |
| 14 | Kanha | KAN |
| 15 | Kharswan | KHA |
| 16 | Kolkata | KOL |
| 17 | Lucknow | LUC |
| 18 | Manas | MAN |
| 19 | Munnar | MUN |
| 20 | Nanded | NAN |
| 21 | Nedumkandam | NED |
| 22 | Ooty | OTY |
| 23 | Pachmarhi | PACH |
| 24 | Patna | PAT |
| 25 | Pune | PUNE |
| 26 | Ranthambore | RAN |
| 27 | Sathyamangalam | SAT |
| 28 | Shillong | SHI |
| 29 | Sirsi | SIR |
| 30 | Tadoba | TAD |
| 31 | Thiruvananthapuram | THI |
| 32 | Tiruchirappalli | TRI |
| 33 | Udaipur | UDA |
| 34 | Visakhapatnam | VIS |
| 35 | Katarniaghat | KAT |
| 36 | Soreng | SIK |

*Table B F_ST_ between different populations of Lantana in India*

Table C AIC values for alternative demographic scenarios tested for both flower colour combinations

| **Demographic Scenario** | **AIC for White pink and Orange** | **AIC for Yellow pink and Orange** |
| --- | --- | --- |
| Sim 1 | 24483.56 | 16458.22 |
| Sim 2 | 24465.26 | 16544.96 |
| Sim 3 | 24427.1 | 17929.05 |
| Sim 4 | 24446.28 | 16537.18 |
| Sim 5 | 24416.66 | 16559.64 |
| Sim 6 | 23581.18 | 17021.21 |
| Sim 7 | 24508.32 | 16461.95 |
| **Sim 8** | **23414.22** | **16364.12** |
| Sim 9 | 24512.84 | 16451.37 |
| Sim 10 | 24468.73 | 16504.92 |

Table D: Estimated demographic parameters under the Sim8 scenario for the orange and white–pink morphs. TBOT – Start of bottleneck backwards in time, TENBOT – end of bottleneck, SplTm – Split time, MIG21 – Migration from 2 to 1, MIG12 – Migration from 1 to 2

| **White-pink and Orange** | | | | | |
| --- | --- | --- | --- | --- | --- |
| **Replication** | **TENDBOT** | **TBOT** | **SplTm** | **MIG21** | **MIG12** |
| 1 | 162 | 97 | 4795 | 0.0189922 | 0.0117474 |
| 2 | 167 | 95 | 4843 | 0.0148309 | 0.0114203 |
| 3 | 164 | 91 | 4794 | 0.0189249 | 0.0164912 |
| 4 | 162 | 91 | 4556 | 0.0157511 | 0.0119116 |
| 5 | 161 | 94 | 4707 | 0.0191866 | 0.0141563 |
| 6 | 163 | 90 | 4716 | 0.01422 | 0.0089296 |
| 7 | 166 | 100 | 4677 | 0.0159457 | 0.0121213 |
| 8 | 165 | 94 | 4663 | 0.014621 | 0.0085649 |
| 9 | 161 | 95 | 4561 | 0.013455 | 0.009591 |
| 10 | 161 | 92 | 4908 | 0.0179659 | 0.0148797 |
| **White-pink and Yellow-pink** | | | | | |
| 1 | 165 | 94 | 4386 | 0.0069143 | 0.0509425 |
| 2 | 166 | 91 | 4664 | 0.0077926 | 0.0633768 |
| 3 | 160 | 93 | 4469 | 0.051668 | 0.0059673 |
| 4 | 161 | 91 | 4685 | 0.0060619 | 0.0503543 |
| 5 | 166 | 91 | 2824 | 0.0085102 | 0.0598156 |
| 6 | 161 | 100 | 3775 | 0.0713767 | 0.0097837 |
| 7 | 163 | 98 | 4548 | 0.0090273 | 0.0719626 |
| 8 | 161 | 98 | 4413 | 0.0607776 | 0.0079988 |
| 9 | 162 | 111 | 4362 | 0.0629504 | 0.0096368 |
| 10 | 163 | 107 | 4450 | 0.0586876 | 0.0092196 |

Table E Summary statistics for the MANOVA

| **Source** | **Df** | **Pillai** | **approx F** | **num Df** | **den Df** | **Pr(>F)** | **Significance** |
| --- | --- | --- | --- | --- | --- | --- | --- |
| Flower_colour | 5 | 3.0662 | 40.273 | 50 | 1270 | < 2.2e-16 | *** |
| Residuals | 259 |  |  |  |  |  |  |
