# Supplementary material for: The population structure of invasive Lantana camara is shaped by its mating system
Source: eLife. 2026 Jul 23;14:RP104988. doi: 10.7554/eLife.104988 (PMC13395457; doi:10.7554/eLife.104988)
Supplement: Peer review history

# Editor report: eLife Assessment

**DOI:** 10.7554/eLife.104988.3.sa0

**Authors:** Alexandre Fournier-Level

**Affiliations:** La Trobe University Australia

**Keywords:** Important, Incomplete

The submission by Praveen et al. reports **important** findings describing the structure of genetic and colour variation in its native range for the globally invasive weed *Lantana camara*. Whilst the importance of the research question and the scale of the sampling is appreciated, the analysis, which is currently **incomplete**, requires further tests to support the claims made by the authors.

# Referee report: Reviewer #1 (Public review)

**DOI:** 10.7554/eLife.104988.3.sa1

Summary:

The authors investigated the population structure of the invasive weed Lantana camara from 36 localities in India using 19,008 genome-wide SNPs obtained through ddRAD sequencing.

Strengths:

The manuscript is well-written, the analyses are sound, and the figures are of great quality.

Weaknesses:

The narrative almost completely ignores the fact that this plant is popular in horticultural trade and the different color morphs that form genetic populations are most likely the result of artificial selection by humans for certain colors for trade, and not the result of natural selfing. Although it may be possible that the genetic clustering of color morphs is maintained in the wild through selfing, there is no evidence in this study to support that. The high levels of homozygosity are more likely explained as a result of artificial selection in horticulture and relatively recent introductions in India. Therefore, the claim of the title that "the population structure.. is shaped by its mating system" is in part moot, because any population structure is in large part shaped by the mating system of the organism, but further misleading because it is much more likely artificial selection that caused the patterns observed.

Update after manuscript was revised by authors:

The authors added a selfing experiment, showing that the wild plants are selfing and not outcrossing, which limits the genetic exchange. This supports their claims, but a link with the horticultural industry is still lacking in the study, and conclusions should still be viewed in the regional context of India rather than globally.

# Referee report: Reviewer #2 (Public review)

**DOI:** 10.7554/eLife.104988.3.sa2

Summary:

The authors performed a series of population genetic analyses in *Lantana camara* using 19,008 genome-wide SNPs data from 359 individuals in India. They found clear population structure that did not show a geographical pattern, and flower color was rather associated with population structure. Excess of homozygosity indicate high selfing rate, which may lead to fixation of alleles in local populations and explain the presence of population structure without a clear geographic pattern. Authors also performed a forward simulation analysis, theoretically confirming that selfing promotes fixation of alleles (higher Fst) and reduction in genetic diversity (lower heterozygosity).

Strengths:

Biological invasion is a critical driver of biodiversity loss, and it is important to understand how invasive species adapt to novel environments despite limited genetic diversity (genetic paradox of biological invasion). *Lantana camara* is one of the hundred most invasive species in the world (IUCN 2000), and the authors collected 359 plants from a wide geographical range in India, where *L. camara* has invaded. The scale of the dataset and the importance of the target species are the strength of the present study. Coalescent-based analysis nicely supports the authors' claim that multiple introductions may have contributed the population structure of this species.

Weaknesses:

The main findings of the SLiM-based simulation were that inbreeding promotes fixation of alleles and reduction in genetic diversity. These are theoretically well known, and such findings themselves are not novel, although it may have become interesting if these findings are quantitatively integrated with their empirical findings in the studied species.

# Author comment: Author response

**DOI:** 10.7554/eLife.104988.3.sa3

**Authors:** P Praveen, Rajesh Gopal, Uma Ramakrishnan

**Affiliations:** National Centre for Biological Sciences - TIFR Bengaluru India; Global Tiger Forum - East of Kailash New Delhi India; National Centre for Biological Sciences, TIFR Bangalore India

The following is the authors’ response to the original reviews.

We sincerely thank the editor and both reviewers for their time and thoughtful feedback on our manuscript. We have carefully addressed all the concerns raised in the responses below and incorporated the suggested revisions into the manuscript.

**Public Reviews:**

**Reviewer #1 (Public review):**

Summary:

The authors investigated the population structure of the invasive weed *Lantana camara* from 36 localities in India using 19,008 genome-wide SNPs obtained through ddRAD sequencing.

Strengths:

The manuscript is well-written, the analyses are sound, and the figures are of great quality.

Weaknesses:

The narrative almost completely ignores the fact that this plant is popular in horticultural trade and the different color morphs that form genetic populations are most likely the result of artificial selection by humans for certain colors for trade, and not the result of natural selfing. Although it may be possible that the genetic clustering of color morphs is maintained in the wild through selfing, there is no evidence in this study to support that. The high levels of homozygosity are more likely explained as a result of artificial selection in horticulture and relatively recent introductions in India. Therefore, the claim of the title that "the population structure.. is shaped by its mating system" is in part moot, because any population structure is in large part shaped by the mating system of the organism, but further misleading because it is much more likely artificial selection that caused the patterns observed.

The reviewer raises the possibility that the observed genetic patterns may have originated through the selection of different varieties by the horticultural industry. While it is plausible that artificial selection can lead to the formation of distinct morphs, the presence of a strong structure between them in the wild populations cannot be explained just based on selection. The observed patterns in the inbreeding coefficient and heterozygosity can indeed arise from multiple factors, including past bottlenecks, selection, inbreeding, and selfing. In the wild, different flower colour variants frequently occur in close physical proximity and should, in principle, allow for cross-fertilization. Over time, this gene flow would be expected to erode any genetic structure shaped solely by past selection. However, our results show no evidence of such a breakdown in structure. Despite co-occurring in immediate proximity, the flower colour variants maintain distinct genetic identities. This suggests the presence of a barrier to gene flow, likely maintained by the species' mating system. Moreover, the presence of many of these flower colour morphs in the native range—as documented through observations on platforms like iNaturalist—suggests that these variants may have a natural origin rather than being solely products of horticultural selection.

While it is plausible that horticultural breeding involved efforts to generate new varieties through crossing—resulting in the emergence of some of the observed morphs—even if this were the case, the dynamics of a self-fertilizing species would still lead to rapid genetic structuring. Following hybridization, just a few generations of selfing are sufficient to produce inbred lines, which can then maintain distinct genetic identities. As discussed in our manuscript, such inbred lines could be associated with specific flower colour morphs and persist through predominant self-fertilization. This mechanism provides a compelling explanation for the strong genetic structure observed among co-occurring flower colour variants in the wild.

To further validate this, we conducted a bagging experiment on *Lantana camara* inflorescences to exclude insect-mediated cross-pollination. The results showed no significant difference in seed set between bagged and open-pollinated flowers, supporting the conclusion that *L. camara* is primarily self-fertilizing in India. These results are included in the revised manuscript.

As the reviewer rightly points out, the mating system of a species plays a crucial role in shaping patterns of genetic structure. However, in many natural populations, structuring patterns are often influenced by a combination of factors such as selection, barriers to gene flow, and genetic drift. In some cases, the mating system exerts a more prominent influence at the microgeographic level, while in others, it can shape genetic structure at broader spatial scales. What is particularly interesting in our study is that - the mating system appears to shape genetic structure at a subcontinental scale. Despite the species having undergone other evolutionary forces—such as a genetic bottleneck and expansion due to its invasive nature—the mating system exerts a more pronounced effect on the observed genetic patterns, and the influence of the mating system is remarkably strong, resulting in a clear and consistent genetic structure across populations.

**Reviewer #1 (Recommendations for the authors):**

*Lantana camara* is a globally invasive plant as the authors mention in their manuscript, but this study only focuses on India. This should be reflected in the title.

The reviewer has suggested that the title should reflect the study area. Since our sampling covers nearly all regions in India, we believe the patterns observed here are likely representative of those in other parts of the invaded range. For this reason, we would prefer to retain the current heading.

It would be helpful if the pictures of the flowers in Figure 3 were larger to more clearly see the different colors.

As per the reviewers suggestion we have increased the size of the images to improve clarity.

Figure 4 could probably be moved to supplemental material, it does not add much to the results.

We feel it is important to reiterate that the patterns we observe in Lantana are consistent with what one would expect in any predominantly self-fertilizing species. It act as an additional proof and therefore, we believe it is important to retain this figure, as it effectively conveys this link.

**Reviewer #2 (Public review):**

Summary:

The authors performed a series of population genetic analyses in *Lantana camara* using 19,008 genome-wide SNPs data from 359 individuals in India. They found a clear population structure that did not show a geographical pattern, and that flower color was rather associated with population structure. Excess of homozygosity indicates a high selfing rate, which may lead to fixation of alleles in local populations and explain the presence of population structure without a clear geographic pattern. The authors also performed a forward simulation analysis, theoretically confirming that selfing promotes fixation of alleles (higher Fst) and reduction in genetic diversity (lower heterozygosity).

Strengths:

Biological invasion is a critical driver of biodiversity loss, and it is important to understand how invasive species adapt to novel environments despite limited genetic diversity (genetic paradox of biological invasion). *Lantana camara* is one of the hundred most invasive species in the world (IUCN 2000), and the authors collected 359 plants from a wide geographical range in India, where *L. camara* has invaded. The scale of the dataset and the importance of the target species are the strengths of the present study.

Weaknesses:

One of the most critical weaknesses of this study would be that the output modelling analysis is largely qualitative, which cannot be directly comparable to the empirical data. The main findings of the SLiM-based simulation were that selfing promotes the fixation of alleles and the reduction of genetic diversity. These are theoretically well-reported knowledge, and such findings themselves are not novel, although it may have become interesting these findings are quantitatively integrated with their empirical findings in the studied species. In that sense, a coalescent-based analysis such as an Approximate Bayesian Computation method (e.g. DIY-ABC) utilizing their SNPs data would be more interesting. For example, by ABC-based methods, authors can infer the split time between subpopulations identified in this study. If such split time is older than the recorded invasion date, the result supports the scenario that multiple introductions may have contributed to the population structure of this species. In the current form of the manuscript, multiple introductions were implicated but not formally tested.

Through our SLiM simulations, we aimed to demonstrate that a pattern of strong genetic structure within a location (similar to what we observed in *Lantana camara*) can arise under a predominantly self-fertilizing mating system. These simulations were not parameterized using species-specific data from *Lantana* but were intended as a conceptual demonstration of the plausibility of such patterns under selfing using SNP data. While the theoretical consequences of self-fertilisation have been widely discussed, relatively few studies have directly modelled these patterns using SNP data. Our SLiM simulations contribute to this gap and support the notion that the observed genetic structuring in *Lantana* may indeed result from predominant self-fertilisation. Therefore, we conducted these simulations ourselves for invasive plants to test whether the patterns we observed are consistent with expectations for a predominantly self-fertilising species.

Additionally, as suggested by the reviewer, we have performed demographic history simulations using fastsimcoal2 to investigate the divergence among different flower colour morphs. The results have been incorporated into the revised manuscript.

First, the authors removed SNPs that were not in Hardy-Weinberg equilibrium (HWE), but the studied populations would not satisfy the assumption of HWE, i.e., random mating, because of a high level of inbreeding. Thus, the first screening of the SNPs would be biased strongly, which may have led to spurious outputs in a series of downstream analyses.

Applying a HWE filter is a common practice in genomic data analysis because it helps remove potential sequencing or genotyping artefacts, which can otherwise bias downstream analyses. However, we understand that HWE filtering can also remove biologically informative loci and potentially bias the analysis, especially when a stringent cutoff is used. A strict filter might retain only loci that perfectly fit Hardy–Weinberg expectations and exclude sites influenced by real evolutionary processes like selection and/or inbreeding.

To balance this, we used a mild HWE filter, aiming to remove clear artefacts while retaining loci that may reflect genuine biological signals. Another reason for applying it is that many downstream tools, for example, admixture, assume the markers are neutral and not strongly deviating from HWE (although this assumption may not always hold). This helps in avoiding the complexity of the model.

Second, in the genetic simulation, it is not clear how a set of parameters such as mutation rate, recombination rate, and growth rate were determined and how they are appropriate.

We have cited the references for these values in the manuscript. However, for Lantana, many such baseline data are not available, so we used general values reported for plants, which is an accepted approach when working with understudied species. Moreover, the aim of these simulations was to develop a general understanding of how mating systems influence genetic diversity in invasive plants, rather than to parameterize the simulations specifically for Lantana.

While we acknowledge that this simulation does not provide an exact representation of the species' evolutionary history, the goal of the simulation was not to produce precise estimates but rather to illustrate the feasibility of such strong genetic structuring resulting from self-fertilisation alone.

Importantly, while authors assume the selfing rate in the simulation, selfing can also strongly influence the effective mutation rate (e.g. Nordborg & Donnelly 1997 Genetics, Nordborg 2000 Genetics). It is not clear how this effect is incorporated in the simulation.

In genetic simulations, it is often best to begin with simpler scenarios involving fewer parameters, and we followed this approach. As the reviewer rightly pointed out, selfing can influence multiple factors such as mutation and recombination rates. However, to first understand the broad effects, we chose to work with simpler scenarios where both mutation and recombination rates were kept constant.

Third, while the authors argue the association between flower color and population structure, their statistical associations were not formally tested.

We thank the reviewer for this valuable suggestion. We have performed a MANOVA to test the association between flower colour and genetic structure. These results are incorporated in the revised manuscript.

Also, it is not mentioned how flower color polymorphisms are defined. Could it be possible to distinguish many flower color morphs shown in Figure 1b objectively?

We carefully considered this and defined our criteria based on flower colour. Specifically, we named morphs according to the colour of both young and old flowers. If both stages shared the same colour, we used that colour as the name. As shown in Figure 1b, it is possible to reliably distinguish between the different flower colour morphs. While one could also measure flower colour using a photometer, we believe both approaches yield similar results.

I am concerned particularly because the authors also mentioned that flower color may change temporally and that a single inflorescence can have flowers of different colors (L160).

The flower colour changes within an inflorescence, with young flowers shifting colour after pollination. However, this trend is consistent within a plant; for example, the yellow–pink morph always changes from yellow to pink. Based on this consistency, we incorporated a naming system that considers both the colour of younger and older flowers.

**Reviewer #2 (Recommendations for the authors):**

Figure 4: Figures a and b are not the "signatures of high inbreeding", because such patterns could also simply happen due to geographical isolation. The title of the figure could be changed. Figure 4c should be presented as a histogram.

We have incorporated this suggestion into the manuscript and revised the figure title accordingly. However, we believe that presenting Figure 4c in its current form is more informative.

L459 "in the introduced range, Lantana is self-compatible": is it self-incompatible in the native range? If it is known, it could be mentioned in the manuscript.

A previous study from India demonstrated that self-fertilisation is possible in Lantana, providing an additional line of evidence for our findings. However, Lantana remains poorly studied in its native range, and to the best of our knowledge, only a single study has examined its pollination biology there, which we have cited in this paper.
